# Protocol of a mixed method, randomized controlled study to assess the efficacy of a psychosocial intervention to reduce fatigue in patients with End-Stage Renal Disease (ESRD)

**DOI:** 10.1186/s12882-016-0277-8

**Published:** 2016-07-08

**Authors:** Wieke E. van der Borg, Karen Schipper, Tineke A. Abma

**Affiliations:** Department of Medical Humanities, VU University Medical Center/EMGO+, De Boelelaan 1089a, 1081 HV Amsterdam, The Netherlands

**Keywords:** Study protocol, End-stage renal disease, Fatigue, Quality of life, Psychosocial, Intervention, Social workers, Randomised controlled trial, Mixed methods, Process evaluation

## Abstract

**Background:**

Patients with end-stage renal disease (ESRD) commonly suffer from severe fatigue, which strongly impacts their quality of life (QoL). Although fatigue is often attributed to disease- and treatment characteristics, research also shows that behavioural, psychological and social factors affect perceived fatigue in dialysis patients. Whereas studies on fatigue in other chronic patient groups suggest that psychological or psychosocial interventions are effective in reducing fatigue, such interventions are not yet available for ESRD patients on dialysis treatment. The objective of this study is to examine the efficacy of a psychosocial intervention for dialysis patients aimed at reducing fatigue (primary outcome) and improving QoL (secondary outcome). The intervention consists of counselling sessions led by a social worker. The implementation process and patients’ and social workers’ expectations and experiences with the intervention will also be evaluated.

**Methods/Design:**

This study follows a mixed-methods design in which both quantitative and qualitative data will be collected. A multi-centre, randomised controlled trial (RCT) with repeated measures will be conducted to quantitatively assess the efficacy of the psychosocial intervention in reducing fatigue and improving QoL in ESRD patients. Additional secondary outcomes and medical parameters will be assessed. Outcomes will be compared to patients receiving usual care. A sample of 74 severely fatigued dialysis patients will be recruited from 10 dialysis centres. Patients will be randomly assigned to the intervention or control group. Outcomes will be assessed at baseline, post intervention/16 weeks, and at three and six-month follow-ups. A qualitative process evaluation will be conducted parallel to/following the effectiveness RCT. Interviews and focus groups will be conducted to gain insight into patients’ and social workers’ perspectives on outcomes and implementation procedures. Implementation fidelity will be assessed by audio-taped and written registrations. Participatory methods ensure the continuous input of experiential knowledge, improving the quality of study procedures and the applicability of outcomes.

**Discussion:**

This is the first mixed method study (including an RCT and qualitative process evaluation) to examine the effect and implementation process of a psychosocial intervention on reducing fatigue and improving QoL in ESRD patients on dialysis treatment.

**Trial registration:**

NTR5366, The Netherlands National Trial Register (NTR), registered August 26, 2015.

**Electronic supplementary material:**

The online version of this article (doi:10.1186/s12882-016-0277-8) contains supplementary material, which is available to authorized users.

## Background

End-stage renal disease (ESRD) is a common chronic illness with an increasing incidence and prevalence [1, 2]. In the Netherlands, over 16,000 patients require renal replacement therapy due to severe kidney failure [3]. Although renal transplantation is the most preferred treatment [4, 5], many patients cannot profit from this option because of the donor shortage [5, 6] or medical ineligibility for receiving a donor kidney [7, 8]. Consequently, many ESRD patients depend on dialysis treatment. In the Netherlands, around 6,500 ESRD patients receive either haemodialysis or peritoneal dialysis treatment [9]. Although dialysis treatment is life-saving, the burdens of treatment are substantial and the therapy only replaces 10–15 % of normal renal function [10]. Dialysis patients experience multiple enduring health problems, of which fatigue is one of the most frequent complaints [11–14]. The prevalence of (severe) fatigue in dialysis patients ranges from 60–97 % [15, 16]. Fatigue is known as a subjective, unpleasant symptom that can vary from tiredness to exhaustion, which interferes with individuals’ ability to function in their normal (physical and mental) capacity [17–19]. Fatigue limits patients’ daily activities and independence, is often perceived as a source of stress and is commonly associated with reduced quality of life (QoL) [12, 13, 17]. Fatigue in patients on dialysis is often attributed to disease and treatment characteristics like anaemia, inflammation, medication, dialysis intensity and frequency. Except for Erythropoietin (EPO) injections to prevent anaemia, no medication is known to affect/reduce feelings of fatigue [20–22]. Although fatigue is often perceived as a physiological side-effect of the chronic illness [12], research shows that fatigue is not only grounded in physiological factors. Fatigue seems to be caused by a complex interaction of biological processes, psychosocial phenomena and behavioural manifestations [19]. Previous research identified various behavioural, psychological and social factors influencing fatigue in long-term dialysis patients such as anxiety, stress, depression, sleep disorders, substance use, physical inactivity and (lack of) social support [12]. Another study also highlighted the complex character of fatigue in ESRD patients [17]. The authors made a distinction between physical, cognitive and affective fatigue, and identified various physiological, sociodemographic, psychological, behavioural and dialysis-related factors to affect those various dimensions of fatigue.

Non-pharmalogical interventions targeted at behavioural, psychological, cognitive and social factors, may potentially be successful in decreasing fatigue in dialysis patients. Studies on fatigue in patients with cancer [23–28], chronic pain [29], chronic fatigue syndrome [30, 31], brain injury [32] and muscular diseases [33, 34], suggest that such psychological or psychosocial interventions are effective in reducing fatigue that is caused by multiple interacting factors. As far as we know, the literature does not describe (evidence-based) psychological or psychosocial interventions to reduce fatigue in ESRD patients. This can be seen as a deficiency, since such interventions may, based on other populations and causes of fatigue, also be effective in reducing the experienced fatigue of patients on dialysis.

The primary objective of this study is to investigate the efficacy of a psychosocial intervention on the reduction of fatigue provided to patients on dialysis treatment, compared to dialysis patients receiving usual care. Secondary objectives are to study the efficacy of the intervention on improving the QoL and to investigate the possible mediating and moderating effects of coping style, illness cognitions and perceptions, catastrophizing thoughts, feelings of depression, feelings of control and mastery, and social support. A randomised controlled trial (RCT) will be conducted to achieve these objectives. Next to the RCT, a process evaluation will be conducted to gain an in-depth understanding of both patients’ and professionals’ expectations of, and experiences with the psychosocial intervention and its implementation. In addition, the degree to which the intervention is implemented as intended (implementation fidelity) will be evaluated [35–37].

## Methods/Design

### Design and setting

**Fig. 1:**
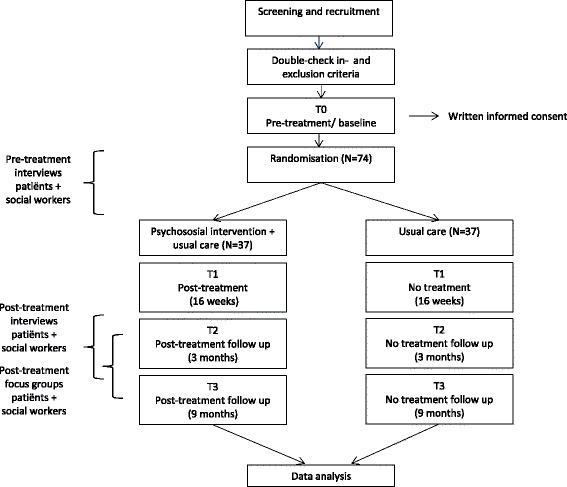
Study design

This study follows a mixed method design in which both quantitative and qualitative methods will be applied for data collection. The study involves a two-arm, multi-centre RCT involving patients with ESRD undergoing a dialysis treatment (either haemodialysis or peritoneal dialysis). Patients will be randomly assigned to either the intervention group (usual treatment and psychosocial counselling by a social worker in the dialysis department, aimed at reducing fatigue) or the control group (usual treatment). Intervention efficacy will be quantitatively measured (repeated measures/self-report questionnaires/medical records) (Fig. 1). A qualitative process evaluation will be conducted to gain insight into patients’ and professionals’ perspectives on outcomes and implementation procedures. Both interviews and focus groups will be applied for data collection. The study will be conducted at both academic and non-academic dialysis centres in the Netherlands. The aim is to include a maximum of 10 centres in total.

### Research team and advisory group

#### Research team

The study will be conducted by a team of three researchers of the VU University Medical Centre (VUmc) and two patient research partners who are experiential experts in the field of kidney diseases/dialysis [38]. One them is a female patient (aged 48) suffering from kidney disease, the second one is a father (aged 66) of a patient who has been on dialysis treatment before transplantation. Both patient research partners are equal members of the research team and will participate in research activities throughout all study phases (research set-up, data collection and data analysis).

The surplus value of involving patient research partners in scientific research has been increasingly acknowledged. The experiential knowledge of patients complements the scientific perspective of academic researchers and contributes to the quality of research in various ways. It helps to assure that research is grounded in relevant clinical needs and targeted to patient-relevant outcomes, it can enhance study design and practicability, it improves data interpretation and it strengthens dissemination [39–42].

#### Advisory group

An advisory group consisting of patients, partners of renal patients and professionals will monitor the study and will be asked for input and feedback throughout all study phases. Participants are five patients/relatives (members of the Kidney Patients Association Netherlands - NVN), two social workers and a nephrologist. The advisory group will contribute to the quality of the study and applicability of outcomes by providing input from both patient and professional perspectives.

### Patient recruitment

In collaboration with the research team, the Dutch Association for Social Workers in Nephrology has informed its members about the opportunity to participate in this multi-centre study. Dialysis centres sign in to participate on a voluntary base. The local recruitment of patients within the participating centres will begin after formal approval is obtained by the head of the department, the local ethics committee and/or the board of directors. The social workers of the participating centres will be involved in the pre-screening and recruitment of patients at their centre (see inclusion and exclusion criteria below). They will be asked to identify all potential participants that fit the trial criteria within their patient population and to (randomly) invite suitable patients to participate in the study. The VUmc research team will provide a flyer and patient information letter to all participating centres in order to assure provision of identical study information to patients. The flyers and letters will be handed to the patients by the local social workers.

If a patient wants to participate, (after verbal consent) contact details will be sent to the first research executive [WB], who will inform patients about the study procedures, answer questions and do a final check on inclusion and exclusion criteria. Subsequently, the first researcher will request that the patients fill out and return the Checklist Individual Strength (CIS-fatigue) questionnaire [43] in order to assess the perceived level of fatigue. If the score is ≥ 35, the final inclusion criterion is met. Patients will be informed about the survey results personally by phone, since we know from a former study that patients do not appreciate written communication about inclusion and exclusion [34]. After obtaining written informed consent and completion of the baseline measurement, participants will be randomly assigned to either the control or intervention group.

#### Sample size

The sample size calculation will be based on examining the difference between the psychosocial intervention versus usual care on the primary outcome ‘subjective experience of fatigue’ (four repeated measurements) [43]. Thirty-seven patients will be needed in each condition, assuming a significance of 5 % (two-tailed) and power of 80 % in detecting a clinically relevant change of eight points on the primary outcome variable [25, 44]. We expect a drop-out rate of 30 % based on an unpublished study by Dialysis Centre Groningen in 2011 (14 % mortality, 5 % kidney transplantation, 11 % remaining).

### Inclusion criteria

- Adult patients (age ≥ 18), male or female, who undergo day dialysis (haemodialysis (HD), Peritoneal Dialysis (PD), at home, a hospital or a dialysis centre);
- Experiencing (severe) fatigue (score CIS-fatigue scale ≥ 35);
- Being able to walk/move for at least 10 min with or without a supporting device such as a walking stick;
- Having a sufficient understanding of the Dutch language in order to participate in counselling, (group) interviews and fill out the questionnaires adequately.

### Exclusion criteria

- Dialysis during the night (since it is assumed that patients on day dialysis experience more severe fatigue compared to patients on night dialysis);
- Participation in other studies or treatments aimed at reducing fatigue;
- Treatment by a psychologist or psychiatrist (for severe psychiatric problems such as depression, psychosis, personality disorders or schizophrenia);
- Alcohol or drug addiction.

Furthermore, social workers will be asked to take into account the dialysis vintage (minimum of three months) and dialysis stability when pre-screening patients for the study.

### Randomisation

Patients who meet the trial criteria and give informed consent to participate in the study will be assigned to either the intervention or the control group. Patients in the control group will receive usual treatment. Patients in the intervention group will receive a protocolled psychosocial intervention provided by a social worker. Computer-generated block randomisation will be performed, ensuring the equal (1:1) allocation of patients to either the intervention or control group at each participating centre. To ensure concealment, the block sizes will not be disclosed. The allocation procedure is performed by an independent research colleague who is not involved in the study. The researcher [WB] will inform patients about the allocation outcome and subsequent study procedures. For ethical reasons, and if desired, patients allocated to the control group will be offered the opportunity to participate in the intervention group after the third measurement. Due to the nature of the intervention neither participants nor social workers can be blinded to allocation. Data entry will be performed by a research assistant who is not informed about group allocation.

### Study intervention

#### Usual care versus psychosocial intervention

Patients in the control and the intervention group will all receive usual care, which may include dialysis treatment, medication and a nutritional regimen. The scope of usual psychosocial care will be assessed at each participating centre. To prevent co-intervention bias, patients participating in other studies or treatments aimed at reducing fatigue will be excluded. Patients will not be restricted in any activities. Any significant changes in health status and/or treatment will be monitored throughout the study.

Patients assigned to the intervention group will receive face to face psychosocial counselling directed to reducing and managing fatigue. Patients will be involved in 4–6 individual protocolled counselling sessions (45 min each) over a period of 16 weeks, and will perform several practical (home) assignments/exercises. The sessions will be given by a local social worker who is already employed at the participating dialysis centre.

#### Intervention protocol

The intervention protocol has been developed by the VUmc research team, of which one of the members works as a health psychologist [KS]. The protocol is partly based on pre-existing interventions that have already been proven effective in the treatment of fatigue in other patient groups [45–47]. Previous research into the perpetuating factors of (chronic) fatigue in patients with chronic diseases in general [18, 19] and kidney diseases in particular [12, 17] also provided valuable input for protocol development, as well as a (yet unpublished) qualitative interview study on the subjective experience of fatigue in ESRD patients. This qualitative study was initiated by the VUmc research team, and was conducted prior to protocol development.

The protocol consists of eight modules, addressing various behavioural, psychological, cognitive and social aspects that influence fatigue. These factors encompass sleep-wake rhythm and sleep hygiene routines, physical activity, energy distribution, dysfunctional cognitions with respect to fatigue, social support (communication, needs and boundaries), catastrophizing thoughts and worrying. Additional file 1 gives an extended overview of the content of the intervention modules.

#### Intervention instruction for social workers

All involved social workers will be visited by a member of the VUmc research team for instruction on how to use the protocol. Prior to the instruction visit, a digital version of the intervention protocol will be provided. The instruction involves a brief explanation of the eight modules, the method of module selection, counselling procedures and the use of registration forms. Social workers will also be informed about background, main aims and study procedures. All participating professionals are experienced in working with intervention protocols and psychosocial counselling. During the study, a member of the research team will be available to answer questions about the protocol and counselling procedures.

#### Intervention procedures

Because of the variability in influencing factors of fatigue in ESRD patients, counselling will be customised to each patient individually. A certain procedure will, however, be followed. After the patient has returned the baseline measurement (see data collection), the social worker will be informed by the research team and commissioned to start the intervention. The social worker will invite the patient to an intake session in which they jointly will assess and decide which modules are most appropriate. A decision aid is provided in the intervention protocol. A maximum of three to four modules will be selected, which will be addressed in 4–6 individual counselling sessions of about 45 min each. Each module will include one or more (take-home) exercise(s). The patient and social worker agree on the timing and location of the subsequent counselling sessions and whether a partner or relative will participate in (a selection) of the sessions.

### Data collection and management

This study will follow a mixed method design in which both quantitative and qualitative data will be collected.

#### Quantitative measurements

To determine the effects of the intervention on the short- and long-term, repeated quantitative measurements will be conducted (four survey measurements over a period of 13 months). These surveys will be completed by the participants in both the intervention and control groups.

The primary outcome will be fatigue severity, measured by a subscale of the CIS-fatigue [43]. The secondary outcome will be quality of life (kidney disease specific), as measured by the KDQOL [48]. Questionnaires on coping style, illness cognitions/perceptions, catastrophizing thoughts, depression, social support and overall perceptions of mastery and control will be included since these outcomes may be potential moderating or mediating factors. In addition, various medical parameters and demographics will be studied as potential modifiers. All outcome measures and assessment instruments are listed in Table 1.

**Table 1:** Measures and instrumentation

	Instrumentation	T0	T1	T2	T3
Primary outcome measures					
Fatigue severity	Checklist Individual Strength (CIS-fatigue) [43]	x	x	x	x
Secondary outcome measures					
Quality of life	Kidney Disease and Quality of Life Short Form (KDQOL-SF) [48]	x	x	x	x
Mediating/moderating variables					
Social support	Social Support Inventory (SSL-I + SSL-D) [58] + subscale Utrechtse Coping Lijst (UCL) [59]	x	x	x	x
Illness cognitions	Illness Cognition Questionnaire (ICQ) [60]	x	x	x	x
Illness perceptions	Illness Perception Questionnaire (IPQ-R) [61]: subscale emotional representation	x	x	x	x
Coping	Cognitive Emotion Regulation Questionnaire (CERQ) [62]	x	x	x	x
Catastrophizing thoughts	Fatigue Catastrophizing Scale (J-FCS) [63, 64]	x	x	x	x
Mastery	Mastery Scale [65]	x	x	x	x
Depression	Patient Health Questionnaire: Depression (PHQ-9) [66]	x	x		
General effect modifiers					
Demographic variables	Self-report: age, gender, education, ethnicity, social status, employment	x			
Medical history and comorbidity	Self-report: treatment, treatment frequency, transplantability	x			
Medical parameters	Medical records: sleep medication, Epo, Hb, Urea, Creatinine, Protein, Albumin, CRP, weight, height	x			x

For each individual in the intervention group, measurements will be obtained prior to the start of the study period (T0 - baseline), immediately after the intervention period of 16 weeks (T1 - post- treatment), three months after the intervention period (T2 – short-term follow-up) and nine months after the intervention period (T3 – long-term follow-up). For patients participating in the control group, measurements will be obtained according to the same time frame (baseline; + 16 weeks; + three months; + six months). (Participants will be able to decide themselves how and when they fill out the questionnaires (online/paper-pencil; at the dialysis centre/at home). The overall duration of the study will be 13 months for each participant (in both intervention and control group).

#### Qualitative process and effect evaluation

Besides quantitatively measuring intervention effects, understanding the determinants of intervention success or failure, and insight into the nature of the intervention delivery is essential [37, 49–51]. Therefore, we will perform a qualitative evaluation of both the process and perceived effects parallel to/following the effectiveness RCT [52].

##### Interviews and focus groups

Individual interviews and focus groups will be conducted to gain insight into patients’ and social workers’ expectations of, and experiences with the intervention.

Approximately 15 patients who are assigned to the intervention group will be invited for a semi-structured interview about attitudes and expectations regarding the intervention (prior to the start of the intervention). After the post-intervention measurement (T1), again, a subset of 15 (different) patients will be invited for an interview to assess their experiences with the intervention. Several additional topics will be explored such as the nature of the relationship with the (medical) social worker, (changed) attitudes regarding fatigue and future health/fatigue expectations. Purposeful, maximum variation selection will be applied in order to explore attitudes from a broad range of participants (taking into account variation in demographics, treatment and treatment history).

Interviews will take 60–90 min and will take place at a time and location that best suits the patients. In addition, 10 patients will be invited to a focus group discussion to share experiences, validate and deepen issues generated from the interviews, and discuss suggestions to improve the protocol. The duration of this focus group will depend on the capacity of the participating patients, but will not exceed 2.5 h (including breaks).

All participating social workers will be interviewed about their expectations regarding the utility and effectiveness of the intervention protocol. After finishing the last intervention session with the last participating patient, they will be interviewed about their experiences and asked to evaluate the intervention protocol. The semi-structured interviews will take place at the dialysis centre or by phone, and will take approximately 30 min. After the study period, 8–10 (medical) social workers will be invited to a two-hour focus group in order to exchange experiences, deepen issues derived from the interviews and discuss potential improvements of the protocol.

Both interviews and focus groups will be guided by a pre-defined topic list, which will be discussed within the VUmc research team and members of the advisory group in advance. After approval of participants, all interviews and focus groups will be audio-taped and transcribed verbatim. Written interview reports will be sent back to interview and focus group participants to validate findings and improve credibility [53, 54].

Gained insights from both interviews and focus groups will be used to improve the intervention protocol and treatment feasibility, and to determine facilitators and barriers to the successful engagement of both patients and professionals.

##### Implementation fidelity

To assess the implementation fidelity of the intervention, all social workers will be asked to audio-tape one of the counselling sessions held with the second or third patient to whom they offer the intervention. In order to collect a variety in recorded sessions they will be asked to record the second or third session, under the pre-condition that patients provide written informed consent for audio-tapings. The research executive will collect all audio-tapings and assess whether the modules are offered according to the protocol guidelines and face to face instruction by using a pre-constructed observation checklist.

All social workers will also be asked to provide general information about the dialysis population at their centre and the total number of patients who are eligible, decline and agree to participate. Social workers will be asked to provide registrations for each participating patient on the number and duration of counselling sessions, the involvement of partners or relatives, drop-out rates and reasons for drop-out. Registration forms will be provided prior to the beginning of the trial.

##### Data management and privacy procedures

All privacy sensitive data (any information relating to an identified or identifiable natural person) will be handled according to the rules of protecting personal data (Personal Data Protection Act/Wet Bescherming Persoonsgegevens). All identifying information of participants will be coded and de-personalised. Data will be stored in a secured project file on the VUmc network. Access to the study data will be restricted to involved research members, who all signed a privacy statement.

Signed informed consent forms and returned coded/anonymized questionnaires will be stored in a locker separately from study records that link to participant identifying codes. Only the first responsible researcher [WB] has access to these documents. Data entry will be performed by a research assistant. Data integrity will be assured by random consistency checks/re-entry of data. Audio taped records of interviews/focus groups will be removed after transcripts are provided. Archived electronic data will be kept for a maximum of ten years, informed consent forms for a maximum of five years, in line with the policy guidelines of the Quality Committee of the EMGO+ institute (http://www.emgo.nl/kc/). Collected data will be processed anonymously in publications and reports, preventing identification of individual participants.

### Data analysis

Descriptive statistics for outcome measures will be presented. A paired *t*-test will be used to investigate statistical significant differences in the effects (primary and secondary outcomes) between intervention and control groups. Statistical significance will be defined as *p* ≤ 0.05. Statistical analyses will be performed using the SPSS system for Windows, version 22.0. A detailed analysis plan will be developed in collaboration with a statistical expert. This plan will also address the analysis of potential effect modifiers and confounders, and further exploratory analyses.

Qualitative data will be subjected to a thematic analysis [55, 56]. Themes recurring from the data (interview and focus group transcripts) will be categorised and coded. The research team will meet on a regular basis and discuss emerging themes throughout the research process. Preliminary findings will be discussed with the members of the advisory group.

The quantitative outcomes will be related to, and integrated with the qualitative findings to determine correspondence and discrepancies in perceived and measured effects. The advisory group will be actively involved in discussing findings and final conclusions.

### Treatment modification

Any adverse events reported by participants or observed by social workers will be monitored. If trial participation would lead to, or worsen, health related problems, continuation and/or ancillary care will be discussed with the patient, (medical) social worker and treating specialist.

## Discussion

As far as we know, this study will be the first (mixed method) study (including an RCT and qualitative process evaluation) to consider the effect of a psychosocial intervention on reducing fatigue and improving QoL in patients with ESRD on dialysis.

This study will have several strengths. First, the mixed method design of this study will allow for the integration of quantitative and qualitative findings, enhancing our understanding of intervention effects and implementation processes. Second, involving an advisory group and patient research partners will contribute to the overall quality of research and practical applicability of outcomes.

Existing psychological or psychosocial interventions to reduce fatigue in other patient groups often comprise cognitive behavioural therapy (CBT) provided by health psychologists/therapists [33, 44]. However, a randomised trial study [57] showed that counselling and CBT were equivalent in effect for patients with chronic fatigue in primary care. The psychosocial intervention in this study will be provided by social workers employed at the patient’s dialysis department. Besides cost efficiency, involving social workers will be the best option for various reasons. Whereas a care relationship between patients and social workers often already exists, and social workers are familiar with patients’ background and illness history, we expect this to be of added value in customising the intervention to each patient individually. We also expect that, for patients, the threshold to participate in counselling sessions with a social worker will be lower than the participation in psychological therapy with a psychologist they are not familiar with.

A potential limitation is that, by involving a various group of social workers to implement the intervention, treatment bias may occur. This will partly be countered by allocating an equal number of participants in both intervention and control groups at each participating centre. Furthermore, potential confounding effects will be explored by the assessment of implementation fidelity and exploring contextual dynamics by interviews with both patients and social workers.

Counselling sessions with a social worker, performing (home) exercises and filling out questionnaires, will require certain effort from patients participating in the intervention group. However, we expect that, in the end, these burdens will outweigh the expected gains of reduced fatigue and improved QoL. Both patient research partners and members of the advisory group will be consulted about their ideas on how to reduce burdens for participants as much as possible and how to promote retention. A member of the advisory board emphasised that it may be difficult to keep patients motivated to change when they are fatigued. She suggested paying extra attention when giving patients feedback on progress and preliminary results of efforts during the intervention period. This comment will be emphasised in the intervention instructions for social workers.

This study will help to gain insight into the effectiveness of a psychosocial intervention on reducing fatigue in ESRD patients and improving their quality of life. If the implementation of the intervention is shown to be effective, the protocol will be made available to hospitals and dialysis centres that are interested in offering psychosocial counselling to their patient population.

## Abbreviations

ESRD, end-stage renal disease; QoL, quality of life

## Additional file

Additional file 1: Intervention modules. A schematic overview in which the content and main aims of each intervention module are described. (DOCX 26 kb)
