# Supplementary material for: Protocol of a mixed method, randomized controlled study to assess the efficacy of a psychosocial intervention to reduce fatigue in patients with End-Stage Renal Disease (ESRD)
Source: BMC Nephrol. 2016 Jul 8;17:73. doi: 10.1186/s12882-016-0277-8 (PMC4939050; doi:10.1186/s12882-016-0277-8)
Supplement: Additional file 1: — Intervention modules. A schematic overview in which the content and main aims of each intervention module are described. (DOCX 26 kb) [file 12882_2016_277_MOESM1_ESM.docx]

**Appendix 1. Intervention modules**

| **Module 1: Sleep-wake rhythm and sleep hygiene routines** | **Aims** |
| --- | --- |
| Irregular sleep-wake rhythm and disturbing sleep hygiene routines can perpetuate fatigue. Patients will be made aware of their disturbed sleep-wake rhythm and sleep hygiene routines. They will be encouraged to adhere to a structured bed-time and wake-up schedule and avoid sleeping during the daytime. | - Gain insight into current sleep-wake rhythm and disturbing sleep hygiene routines. - Maintain a regular sleep-wake pattern. - Apply good sleep hygiene practices. |
| **Module 2: Physical activity** |  |
| Feeling low in energy may contribute to an inactive lifestyle. Where inactivity can lead to feeling mentally tired and miserable, activity and physical exertion can be boosting and satisfying. Patients will be guided to gain insight into their activity level, and encouraged to increase physical activity. | - Gain insight into current physical activity level. - Increase physical activity levels. |
| **Module 3: Energy distribution** |  |
| Patients often have a limited amount of energy. Unknowingly, much time is spent on activities that cost a lot of energy: the so-called energy drainers. Often, far less energy is invested in things that provide energy in return. Gaining insight into behaviour/activities and time spent on energy drainers and providers helps to distribute available energy more effectively. | - Gain insight into energy drainers and energy providers. - Gain insight into own behaviour patterns. - Improve energy/activity distribution. |
| **Module 4: Living up to values** |  |
| Fatigue and intense treatment can distract patients from doing what they consider important in life. This module helps them to think about what is valued in life and what can still be achieved despite having a chronic disease. | - Gain insight into own values. - Act in accordance with what is valued in life. |
| **Module 5: Hands on and acceptance** |  |
| In general, people are often tempted to pay a lot of attention to (negative) matters that cannot be changed, for example, by continuously talking or worrying about the issue. On the other hand, there are also (negative) matters that can be resolved, but not acted upon. This module helps to reflect on whether negative matters can be influenced or not. If yes, what are options to take action? If not, how to accept and let go? | - Gain insight into (negative) matters in daily life that can be changed or influenced. - Gain insight into (negative) matters in daily life that cannot be changed or influenced. - Act upon what can be changed or influenced. - Accept what cannot be changed or influenced. |
| **Module 6: Dysfunctional cognitions** |  |
| Cognitions strongly influence how we feel and what we do. Dysfunctional (negative) cognitions can be energy draining and increase feelings of fatigue. Helpful (positive) cognitions can have the opposite effect. Explaining this effect can help to reduce dysfunctional cognitions. The patient explores how to convert dysfunctional cognitions into positive thinking. | - Gain insight into the energy draining effect of catastrophizing thoughts. - Transform negative thinking into helpful thoughts. |
| **Module 7: Social support (communication, needs and boundaries)** |  |
| Fatigue is often invisible to others. As a consequence, the social environment often lacks understanding about how impactful the (side effects of) kidney disease and the treatment may be. Patients may feel misunderstood, which is often related to ineffective communication about needs and constraints. This module helps to discover whether patients hold (un)realistic expectations of others, and whether there is a discrepancy between actual support and desired support. The patient gains insight into his/her social network, the therapist helps to instil better communication and more realistic expectations toward the patient’s social support group. | - Gain insight into current state of (social) support and support needs. - Managing better communication with social network (needs and boundaries). |
| **Module 8: In control of worrying** |  |
| Worrying can be very tedious and may disturb sleep. This module helps patients to become aware of the impact of worrying and tries to give patients more control over their thoughts, declining the impact and frequency of worrying. | - Gain insight into frequency and impact of worrying thoughts. - Gain (more) control over worrying thoughts. |
